# Frequency and management of maternal infection in health facilities in 52 countries (GLOSS): a 1-week inception cohort study

**DOI:** 10.1016/S2214-109X(20)30109-1

**Published:** 2020-04-27

**Authors:** The WHO Global Maternal Sepsis Study (GLOSS) Research Group, Mercedes Bonet, Vanessa Brizuela, Edgardo Abalos, Cristina Cuesta, Adama Baguiya, Mónica Chamillard, Bukola Fawole, Marian Knight, Seni Kouanda, Pisake Lumbiganon, Ashraf Nabhan, Ruta J Nadisauskiene, Abdulfetah Abdulkadir, Richard MK Adanu, Mohammad Iqbal Aman, William E. Arriaga Romero, Bouchra Assarag, Kitty W.M. Bloemenkamp, Aigul Boobekova, Mihaela A. Budianu, Vicenç Cararach, Rigoberto Castro, Sylvia Cebekhulu, José Guilherme Cecatti, Lotte Berdiin Colmorn, Ala Curteanu, Serena Donati, Hla Mya Thway Einda, Yasser Salah El Deen, Faysal El Kak, Mohamed Elsheikh, Maria F Escobar-Vidarte, Marisa Mabel Espinoza, María Ester Estrada, Luis Aaron Gadama, Sourou B Goufodji, Saima Hamid, Rosalinda Hernandez Munoz, Nazarea Herrera Maldonado, Kapila Jayaratne, Saule Kabylova, Alexandra Kristufkova, Vijay Kumar, David Lissauer, Wilson Mereci, Meile Minkauskiene, Philippe Moreira, Stephen Munjanja, Nafissa B. Osman, Henri Gautier Ouedraogo, Aquilino M. Perez, Julia Pasquale, Lucian Puscasiu, Zahida Qureshi, Zenaida Recidoro, Carolina C. Ribeiro-do-Valle, Dhammica Rowel, Hamadoun Sangho, Amir Babu Shrestha, Thitiporn Siriwachirachai, Pierre Marie Tebeu, Khaing Nwe Tin, Dinh Anh Tuan, Rathavy Tung, Griet Vandenberghe, Buyanjargal Yadamsuren, Dilrabo Yunusova, Nelly Zavaleta Pimentel, Bashir Noormal, Virginia Díaz, Charlotte Leroy, Kristien Roelens, M. Christian Urlyss Agossou, Christiane Tshabu Aguemon, Patricia Soledad Apaza Peralta, Víctor Conde Altamirano, Vincent Batiene, Kadari Cisse, Kannitha Cheang, Phirun Lam, Elie Simo, Emah Irene Yakana, Javier Carvajal, Paula Fernández, Jens Langhoff-Roos, Paola Vélez, Alaa Sultan, Alula M. Teklu, Dawit Worku, Philip Govule, Charles Noora Lwanga, María Guadalupe Flores Aceituno, Carolina Bustillo, Bredy Lara, Vanita Suri, Sonia Trikha, Irene Cetin, Carlo Personeni, Guldana Baimussanova, Balgyn Sagyndykova, George Gwako, Alfred Osoti, Raisa Asylbasheva, Damira Seksenbaeva, Saad Eddine Itani, Sabina Abou Malham, Diana Ramašauskaitė, Owen Chikhwaza, Eddie Malunga, Haoua Dembele, Hamadoun Sangho, Fanta Eliane Zerbo, Filiberto Dávila Serapio, Juan I. Islas Castañeda, Tatiana Cauaus, Victor Petrov, Seded Khishgee, Bat-Erdene Lkhagvasuren, Amina Essolbi, Rachid Moulki, Zara Jaze, Arlete Mariano, Thae Maung Maung, Tara Gurung, Sangeeta Shrestha, Marcus J. Rijken, Thomas Van Den Akker, María Esther Estrada, Néstor J. Pavón Gómez, Olubukola Adesina, Chris Aimakhu, Rizwana Chaudhri, M. Adnan Khan, María del Pilar Huatuco Hernández, Maria Lu Andal, Carolina Paula Martin, Léopold Diouf, Dembo Guirassy, Miroslav Borovsky, Ladislav Kovac, Laura Cornelissen, Priya Soma-Pillay, Marta López, María José Vidal Benedé, Hemali Jayakody, Mohamed Elsheikh, Wisal Nabag, Sara Omer, Victoria Tsoy, Urunbish Uzakova, Thumwadee Tangsiriwatthana, Catherine Dunlop, Jhon Roman, Gerardo Vitureira, Luong Ngoc Truong, Nghiem Thi Xuan Hanh, Mugove Madziyire, Thulani Magwali, Linda Bartlett, Fernando Bellissimo-Rodrigues, Shevin T. Jacob, Sadia Shakoor, Khalid Yunis, Liana Campodónico, Hugo Gamerro, Daniel Giordano, Fernando Althabe, A. Metin Gülmezoglu, João Paulo Souza

## Abstract

**Background:**

Maternal infections are an important cause of maternal mortality and severe maternal morbidity. We report the main findings of the WHO Global Maternal Sepsis Study, which aimed to assess the frequency of maternal infections in health facilities, according to maternal characteristics and outcomes, and coverage of core practices for early identification and management.

**Methods:**

We did a facility-based, prospective, 1-week inception cohort study in 713 health facilities providing obstetric, midwifery, or abortion care, or where women could be admitted because of complications of pregnancy, childbirth, post-partum, or post-abortion, in 52 low-income and middle-income countries (LMICs) and high-income countries (HICs). We obtained data from hospital records for all pregnant or recently pregnant women hospitalised with suspected or confirmed infection. We calculated ratios of infection and infection-related severe maternal outcomes (ie, death or near-miss) per 1000 livebirths and the proportion of intrahospital fatalities across country income groups, as well as the distribution of demographic, obstetric, clinical characteristics and outcomes, and coverage of a set of core practices for identification and management across infection severity groups.

**Findings:**

Between Nov 28, 2017, and Dec 4, 2017, of 2965 women assessed for eligibility, 2850 pregnant or recently pregnant women with suspected or confirmed infection were included. 70·4 (95% CI 67·7–73·1) hospitalised women per 1000 livebirths had a maternal infection, and 10·9 (9·8–12·0) women per 1000 livebirths presented with infection-related (underlying or contributing cause) severe maternal outcomes. Highest ratios were observed in LMICs and the lowest in HICs. The proportion of intrahospital fatalities was 6·8% among women with severe maternal outcomes, with the highest proportion in low-income countries. Infection-related maternal deaths represented more than half of the intrahospital deaths. Around two-thirds (63·9%, n=1821) of the women had a complete set of vital signs recorded, or received antimicrobials the day of suspicion or diagnosis of the infection (70·2%, n=1875), without marked differences across severity groups.

**Interpretation:**

The frequency of maternal infections requiring management in health facilities is high. Our results suggest that contribution of direct (obstetric) and indirect (non-obstetric) infections to overall maternal deaths is greater than previously thought. Improvement of early identification is urgently needed, as well as prompt management of women with infections in health facilities by implementing effective evidence-based practices.

**Funding:**

UNDP–UNFPA–UNICEF–WHO–World Bank Special Programme of Research, Development and Research Training in Human Reproduction, WHO, Merck for Mothers, and United States Agency for International Development.

## Introduction

Maternal infections are an important cause of maternal mortality and severe maternal morbidity.^1^, ^2^ Global estimates suggest that direct (obstetric) infections are the third most common cause of maternal mortality, representing about 10·7% of maternal deaths,^1^ with the largest toll estimated in low-income and middle-income countries (LMICs) at 10·7% compared with high-income countries (HICs) at 4·7%.^1^ The contribution of infections to maternal deaths could be larger, as these figures do not include deaths due to abortion-related infections or indirect (non-obstetric) infections, which are not a result of, but aggravated by, pregnancy. Maternal deaths due to infection occur mainly through maternal sepsis, “a life-threatening condition defined as organ dysfunction resulting from infection during pregnancy, childbirth, post-abortion, or post-partum period”.^3^ This definition aligns with the recent Sepsis-3 definition for adults^4^ and includes both direct and indirect infections.^5^, ^6^

Accurate assessment of the burden of maternal infections and its complications, including sepsis, is challenging given differences in case definitions and the populations studied. This assessment is further complicated by the physiological changes during pregnancy that not only predispose women to and aggravate their response to infection, but also complicate its identification and management.^7^ Few studies have reported maternal infections across the continuum of pregnancy to post-partum or post-abortion.^8^ The Global Burden of Disease study estimated that 11·9 million cases of direct maternal infections occurred in 2017.^9^ Generally, data for maternal sepsis in LMICs are scarce. The latest estimates on global burden of sepsis suggest that maternal disorders complicated with sepsis reached 5·7 million cases globally in 2017.^10^ Data from the 1990s suggested an incidence of 1–2 cases per 1000 livebirths.^11^ Studies from the early-2000s, mainly from HICs, reported lower incidences of 0·1–0·6 per 1000 deliveries per year.^7^, ^8^, ^12^

**Research in context**

**Evidence before this study**

We identified primary studies and systematic reviews on frequency and management of maternal infections and sepsis using results of a previous systematic review by us, including searches in PubMed, MEDLINE, and Embase from Jan 1, 2010, to Feb 15, 2016, with no language restrictions (updated in September, 2019). We also identified WHO publications on the topic, and checked reference lists to identify additional studies. Globally, direct (obstetric) maternal infections are the third most common cause of maternal mortality, representing about 10·7% of all maternal deaths. Infections are also an important cause of indirect (non-obstetric) maternal deaths (eg, malaria, HIV, and influenza-like illness, among others). Globally, in 2017, there were approximately 5·7 million women presenting with maternal disorders complicated with sepsis. The reported incidence of maternal sepsis varies across settings from 0·1 to 2·0 cases per 1000 livebirths. However, the true burden of maternal sepsis and its complications is uncertain given the absence of standard definitions, identification criteria, and measurement tools, as well as variations in populations and sources of infections studied. It is globally recognised that prevention, early diagnosis, and prompt management of infections and sepsis are key factors for reducing related morbidity and mortality, as reflected in the 2017 World Health Assembly sepsis resolution.

**Added value of this study**

To the best of our knowledge, this is the first study to provide global data for the frequency and intrahospital management of maternal infections and its complications in 713 health facilities in 52 countries, across the continuum of pregnancy and post-pregnancy timelines. This study provides insights on frequency of maternal infections, according to demographic, obstetric, clinical characteristics and outcomes, and coverage of core practices for the prevention, early identification, and management of maternal infections.

**Implications of all the available evidence**

Maternal direct and indirect infections are an important underlying and contributing cause of maternal mortality and severe morbidity. Improved understanding of epidemiological and clinical characteristics of maternal infections is key for sustaining the reduction of preventable maternal morbidity and mortality. To do so, further efforts are required for the development and implementation of comprehensive approaches for effective prevention, improved identification, monitoring, and management of maternal infections and sepsis in health facilities.

The prevention, early diagnosis, and prompt management of sepsis are key factors for reducing related morbidity and mortality, as reflected in the 2017 World Health Assembly resolution on sepsis.^13^ Several interventions, including trigger tools, appropriate use of antibiotics, and patient-care bundles improve monitoring and reduce infection-related deaths and severe morbidity, both in the general^14^ and the obstetric populations.^15^, ^16^, ^17^ However, data as to how well these interventions are followed and how maternal infections are identified and managed in health facilities are scarce. This information is key for the implementation of quality improvement programmes and the reduction of preventable infection-related maternal deaths and severe morbidities.

In 2017, WHO led the Global Maternal Sepsis Study (GLOSS) and Awareness Campaign in health facilities from 52 countries, under the umbrella of the “Global Maternal and Neonatal Sepsis Initiative”^3^ and in response to the World Health Assembly resolution on sepsis.^13^ The aim of the study was to improve understanding of the epidemiology and predictors of maternal infections and sepsis in health facilities, as well as current management and associated factors. We report here the main findings of GLOSS on frequency of maternal infections, according to demographic, obstetric, clinical characteristics, and outcomes, as well as coverage of core practices for early identification and management of maternal infections.

## Methods

### Study design and participants

**Figure 1:**
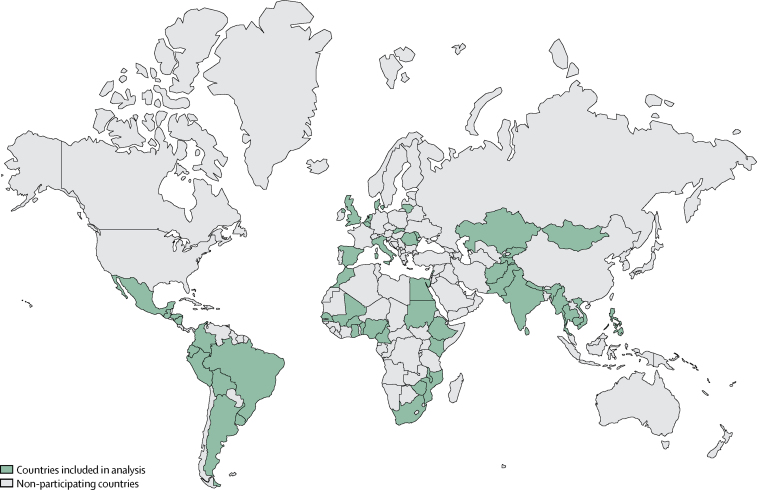
Countries participating in the global maternal sepsis study Eligible health facilities in purposively selected geographical areas in each country participated in the study. The boundaries shown on this map do not imply the expression of any opinion whatsoever on the part of WHO concerning the legal status of any country, or concerning the delimitation of its frontiers or boundaries.

We did a facility-based, prospective, 1-week inception cohort study in selected health facilities in 52 LMICs and HICs (figure 1). We identified all women with suspected or confirmed infection, during any stage of pregnancy and up to the 42nd day after end of pregnancy, admitted to or already hospitalised for at least 12 h in participating health facilities between Nov 28, and Dec 4, 2017, in purposively selected countries and geographical areas, based on prespecified criteria.

Women who had a clinical suspicion or diagnosis of infection, a request for culture of any body fluid, or who were receiving antimicrobial treatment were eligible for inclusion (appendix p 1). All maternal deaths that occurred during the identification week, regardless of the cause, were also included. Women who presented with non-severe, localised, uncomplicated infection; uncomplicated chronic infection; bacterial colonisation; non-infectious hypothermia or hyperthermia; or who received prophylactic antibiotics were not eligible as defined in the appendix (p 1). Women were followed up for up to 6 weeks or until hospital discharge, transfer to a health facility outside the study area, or death, whichever occurred first and regardless of pregnancy outcome. Nine women remained hospitalised at the end of the follow-up period, and their outcomes were collected at the time of discharge. Infants born to women included in the study were followed up for 7 days after birth or until hospital discharge, transfer outside the participating area, or death, whichever occurred first.

All health facilities providing obstetric, midwifery, or post-abortion care; or facilities with an emergency room, adult ward, intensive care unit or high dependency care; or any other setting where pregnant or recently pregnant women could be admitted because of pregnancy-related complications located within the selected geographical areas were eligible and invited to participate.

Ethical approvals were obtained from the WHO ethics review committee and as required by national or local entities. Women were informed about the study using posters placed in visible areas of the participating health facilities. In addition, study teams informed all eligible women about the study and the need to review their medical records for this purpose, as well as those of their neonates. Written informed consent or a waiver of written consent (opt-out) was obtained as required by local or national committees.

Details of the study protocol, including selection of countries, geographical areas, and facilities, have been published elsewhere.^18^ An awareness campaign targeting health providers accompanied the study.^19^

### Procedures

Data were collected at the area, facility, and individual level using standardised paper forms specially designed for the study. These forms were based on tools used in previous multi-country surveys and existing facility assessment tools, and were customised and piloted for this study. Forms were translated from English into French, Portuguese, Russian, and Spanish, as well as additional official country languages by professional translators as needed. Data collection was standardised wherever possible and defined in the manual of operations designed for this study. A customised data entry and monitoring system was developed for the study.

A single geographical area questionnaire was completed by country teams to collect information on the main characteristics of the area, including estimated population size and number of births (or deliveries) in 2016. In each facility, a one-off facility questionnaire collected information on structural characteristics of each of the participating facilities: type of administration, location, level of specialisation, number of births in 2016 and during the week of identification, and availability of maternity services and adult intensive care unit or high dependency care.

The individual data form collected information on demographic, obstetric, and clinical characteristics of the woman; characteristics of infections and management during stay in the health facility; and pregnancy, maternal, and neonatal outcomes. Infections could be confirmed using clinical examination alone, or complemented by radiological, laboratory, or microbiological findings. Suspicion or confirmation of infection was undertaken as part of standard routine care in health facilities, and the study did not require additional collection of any laboratory, diagnostic, or other investigations. Abortion included any abortive outcome (induced abortion, miscarriage, ectopic, and molar pregnancy) as defined locally. Near-miss criteria (defined as a woman who nearly died but survived a life-threatening condition during pregnancy, childbirth, or post-partum or post-abortion periods) were not collected in six European countries (Belgium, Denmark, Italy, Spain, the Netherlands, the UK) as they implemented an adapted protocol using existing systems of surveillance of maternal morbidity. Inclusions were checked against hospital records, admissions to intensive care units or high dependency care, and for all maternal deaths during the identification week. Data quality assurance processes, including checks for accuracy and completeness of data, were put in place during data collection, data entry, and analysis.

### Statistical analysis

A sample size of 2800 women was estimated to ensure at least 100 cases of severe maternal outcomes (ie, death or near-miss), based on a global birth rate of 19·6 livebirths per 1000 population per year, and assuming a 7% frequency of infections requiring hospital admission. Approximately 50 geographical areas with 2 000 000 inhabitants would have to be included to cover about 40 000 births in 1 week.^18^

Women with infection were assigned to three groups according to the severity of the infection during hospital stay: (1) infection-related severe maternal outcomes included women presenting with WHO near-miss criteria to define organ system dysfunction^2^ or maternal death,^5^ and corresponds to the prespecified primary outcome of the study; (2) infections with complications included women with an invasive procedure to treat the source of infection (vacuum aspiration, dilatation and curettage, wound debridement, drainage [incision, percutaneous, culdotomy], laparotomy and lavage, other surgery), admission to intensive care unit or high dependency care, or transfer to another facility. This group constitutes a composite of prespecified secondary outcomes; (3) and the remaining were classified as less severe infections. We considered women with infection-related severe maternal outcomes as a proxy for maternal sepsis,^3^ which includes infection associated with life-threatening organ dysfunction or failure.

We estimated overall ratios of maternal infection (suspected or confirmed infections) and ratios of infection-related severe maternal outcomes (ie, death or near-miss) per 1000 livebirths in health facilities in 2016, and proportion of intrahospital fatalities among women with severe maternal outcomes, by country income, using the 2018 World Bank classification. We calculated the distribution of maternal demographic, obstetric and clinical characteristics, complications, and outcomes by severity group. We calculated coverage of a core set of practices for early identification and management of maternal infections, including recording of a complete set of vital signs on day of suspicion or diagnosis of infection, early initiation of therapeutic antibiotics or other antimicrobials (initiated the same day of suspicion or diagnosis, or day after if suspicion or diagnosis was after 1800 h), drawing of any samples for culture before initiation of antibiotic therapy, and identification and control of the source of infection.

Maternal deaths without infections that occurred during the identification week were excluded from this analysis (n=20). Missing values were less than 10% for all sociodemographic variables, except schooling (54% of values missing), and less than 5% for all obstetric and other clinical characteristics and outcomes, except anaemia during pregnancy (33% missing), and neonatal status at end of follow-up (12%). Therefore, no additional analyses were undertaken to account for missing data. Two separate manuscripts are being developed to report on additional predefined primary and secondary outcomes, including identification criteria of severe maternal infection and sepsis, and the full set of neonatal outcomes.

Categorical variables are presented as proportions and continuous variables as medians and IQRs. 95% CIs for ratios were calculated using normal approximation. Comparisons between infection severity groups were obtained using ordinal multinomial mixed models for percentages and linear models for medians, adjusting for clustering at the country level. Statistical analyses were done using SAS version 9.4.

### Role of the funding source

The funders of the study had no role in data collection, data analysis, data interpretation, or writing of the report. The corresponding author had full access to all the data in the study and had final responsibility for the decision to submit for publication.

## Results

**Figure 2:**
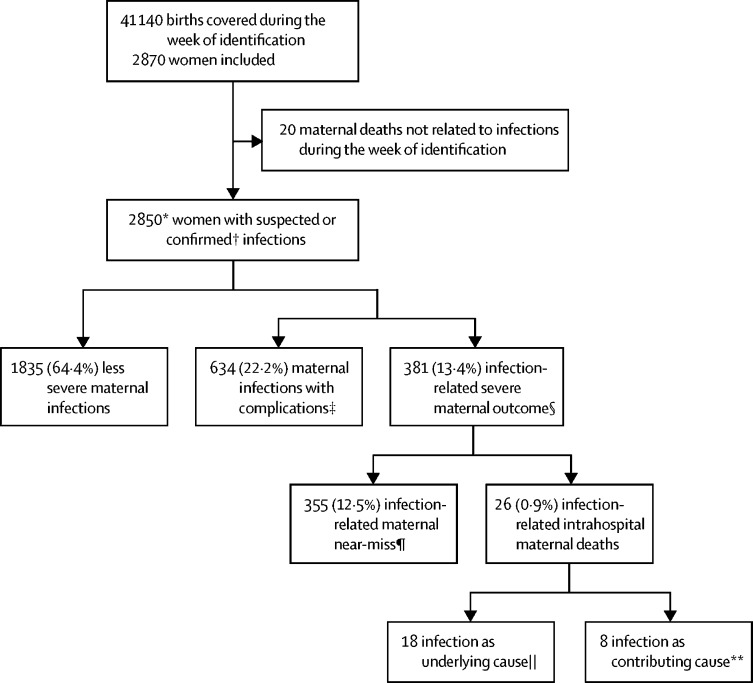
Study profile Percentages are shown as n of total sample. There were 713 health facilities in 52 countries. *2580 women included using full protocol, 290 women included using modified protocol in western European countries (Belgium, Denmark, Italy, Spain, the Netherlands, the UK). †Source of infection clinically, radiologically, or microbiologically confirmed. ‡Includes women who had an invasive procedure to treat the source of infection (vacuum aspiration, dilatation and curettage, wound debridement, drainage [incision, percutaneous, culdotomy], laparotomy and lavage, other surgery), admission to intensive care or high dependency unit, or transfer to another facility. §Maternal death or near-miss. ¶At least one WHO near-miss criteria. ||Includes seven deaths due to direct (obstetric) cause, five due to abortion, six due to indirect (non-obstetric) cause (respiratory infection, meningitis, gastrointestinal). **Includes two deaths due to obstetric haemorrhage, one hypertensive disorder, one other direct cause, two indirect cause, two with unknown cause.

Of 2965 women assessed for eligibility, 2850 women were included in this analysis who were admitted for or already hospitalised with a suspected or confirmed infection (figure 2) in 713 facilities in 52 countries (408 facilities in 43 LMICs and 305 facilities in nine HICs). Participating facilities were mainly public, in urban locations, and tertiary or secondary level (appendix p 2). One participating low-income country was excluded from the study because we were unable to complete the prespecified data quality assurance process (six health facilities, 76 women). Six facilities in LMICs and 16 in HICs in the predefined geographical areas refused to participate. 19 eligible women refused to participate.

The ratio of intrahospital maternal infections (suspected or confirmed) was 70·4 (95% CI 67·7–73·1) women per 1000 livebirths (table 1) and 10·9 (9·8–12·0) of 1000 livebirths presented with infection-related severe maternal outcomes. The highest ratio was observed in upper-middle-income countries (106·4, 95% CI 98·1–114·7) and the lowest in HICs (38·6, 34·1–43·1; table 1). Differences across LMICs were less marked for infection-related severe maternal outcomes.

**Table 1:** Study participants and ratios of maternal infection by country income level*

	**Overall**	**Low-income**	**Lower-middle-income**	**Upper-middle-income**	**High-income**
Countries	52	10	22	11	9
Livebirths in geographical areas in 2016	2 974 356	705 003	1 306 181	481 717	481 455
Women who had a maternal infection*	2850	484	1239	743	384
Women who had a maternal infection with complications†	634	123	307	147	57
Women who had an infection-related severe maternal outcomes‡	381	93	192	92	4
Ratio of maternal infection per 1000 livebirths	70·4 (67·7–73·1)	70·6 (64·1–77·0)	71·6 (67·5–75·6)	106·4 (98·1–114·7)	38·6 (34·1–43·1)
Ratio of infection-related severe maternal outcomes per 1000 livebirths‡§	10·9 (9·8–12·0)	15·1 (12·0–18·2)	12·5 (10·8–14·3)	15·0 (11·8–18·3)	0·6 (0·0–1·1)
Ratio of infection-related maternal near-miss per 1000 livebirths§	10·2 (9·1–11·2)	13·1 (10·3–16·0)	11·7 (10·0–13·4)	14·9 (11·7–18·0)	0·6 (0·0–1·1)

Data are n or ratio (95% CI). Country income level is based on the World Bank country income classification, 2018.

* Suspected or confirmed infection.

† Includes women who had an invasive procedure to treat the source of infection (vacuum aspiration, dilatation and curettage, wound debridement, drainage [incision, percutaneous, culdotomy], laparotomy and lavage, other surgery), admission to intensive care or high dependency unit, or transfer to another facility. Source of infection clinically, radiologically or microbiologically confirmed.

‡ Maternal death or near-miss.

§ At least one WHO near-miss criteria. Geographical areas in six western European countries (Belgium, Denmark, Italy, Spain, the Netherlands, the UK) did not collect data on WHO near-miss criteria.

Intrahospital case fatalities among women with infection-related severe maternal outcomes was 7% (26 of 381 women with severe maternal outcomes; figure 2). Higher case fatalities were found in low-income (12 [15%] of 81) and lower-middle-income (13 [7%] of 179) than in upper-middle-income (one [1%] of 91) countries. No maternal deaths were reported in HICs. Infection was the underlying cause—including direct and indirect infections—or contributing cause in more than half of the intrahospital deaths that occurred in participating facilities during the identification week (19 of 39 maternal deaths; appendix p 3). Additional details of the distribution of organ dysfunction by system, causes of maternal deaths, and contribution of infections to maternal deaths are described in the appendix (pp 3–4).

Table 2 shows demographic, obstetric, and clinical characteristics of all women, and by infection severity groups. At eligibility, more than half of the women were post-partum or post-abortion and were identified at admission to the facility from home. Around a third were identified during pregnancy, not in labour, or were already hospitalised. Sociodemographic characteristics did not vary across the infection severity groups. Number of previous births, identification at post-partum or post-abortion, and anaemia during pregnancy increased with severity of the infection.

**Table 2:** Demographic, obstetric, and clinical characteristics of women who had maternal infections by severity group

		**All women (n=2850)**	**Less severe infections (n=1835)**	Infections with complications*(n=634)	**Infection-related severe maternal outcome**†**(n=381)**	**p value**‡
Age, years		..	..	..	..	<0·0009
	<20	365 (12·9%)	245 (13·4%)	75 (11·9%)	45 (11·9%)	..
	20–35	2104 (74·2%)	1357 (74·4%)	474 (75·0%)	273 (72·2%)	..
	>35	365 (12·9%)	222 (12·2%)	83 (13·1%)	60 (15·9%)	..
Living with partner or spouse		2332 (88·1%)	1507 (88·1%)	511 (87·8%)	314 (88·7%)	0·78
Schooling, years		..	..	..	..	0·055
	<5	174 (13·2%)	102 (12·4%)	40 (13·2%)	32 (16·6%)	..
	5–8	327 (24·7%)	202 (24·5%)	79 (26·0%)	46 (23·8%)	..
	9–11	450 (34·0%)	275 (33·3%)	110 (36·2%)	65 (33·7%)	..
	>11	371 (28·1%)	246 (29·8%)	75 (24·7%)	50 (25·9%)	..
Number of previous births		..	..	..	..	<0·0001
	0	1250 (44·5%)	867 (47·9%)	264 (42·4%)	119 (31·6%)	..
	1–2	1096 (39·0%)	689 (38·1%)	241 (38·7%)	166 (44·1%)	..
	>2	463 (16·5%)	254 (14·0%)	118 (18·9%)	91 (24·2%)	..
Pregnancy status at the time of infection suspected or confirmed§		..	..	..	..	<0·0001
	Pregnant, not in labour	964 (33·9%)	749 (40·8%)	109 (17·2%)	106 (27·9%)	..
	Pregnant, in labour	369 (13·0%)	303 (16·5%)	38 (6·0%)	28 (7·4%)	..
	Post-partum	1246 (43·8%)	711 (39·1%)	347 (52·8%)	188 (49·5%)	..
	Post-abortion¶	269 (9·5%)	71 (3·9%)	140 (22·1%)	58 (15·3%)	..
Location at the time of infection suspected or confirmed§		..	..	..	..	<0·0001
	Arriving from home	1464 (51·5%)	985 (53·9%)	339 (53·6%)	140 (36·8%)	..
	Transferred from another facility	382 (13·4%)	194 (10·6%)	86 (13·6%)	102 (26·8%)	..
	Already hospitalised, non-intensive care unit	926 (32·6%)	650 (35·5%)	169 (26·7%)	107 (28·2%)	..
	Already hospitalised in intensive care unit or high-dependency unit	70 (2·5%)	0 (0·0%)	39 (6·2%)	31 (8·2%)	..
Other complications						
	Anaemia during pregnancy, Hb <11 g/dL	799 (36·3%)	451 (32·6%)	190 (37·4%)	158 (51·1%)	<0·0001
	Pregnancy-related hypertension	296 (11·6%)	134 (8·5%)	77 (12·9%)	85 (22·3%)	<0·0001
	Pre-existing medical condition	169 (6·6%)	69 (4·4%)	38 (6·3%)	62 (16·3%)	<0·0001
	Post-partum haemorrhage‖	229/1861 (12·3%)	106/1184 (9·0%)	49/417 (11·8%)	74/260 (28·5%)	<0·0001
	Obstructed labour or dystocia‖	117/1633 (7·2%)	62/986 (6·3%)	33/387 (8·5%)	22/260 (8·5%)	0·12
	Abortion-related haemorrhage¶	127/322 (39·4%)	23/97 (23·7%)	65/158 (41·1%)	39/67 (58·2%)	<0·0001

Data are n/N (%).

* Includes women who had an invasive procedure to treat the source of infection (vacuum aspiration, dilatation and curettage, wound debridement, drainage [incision, percutaneous, culdotomy], laparotomy and lavage, other surgery), admission to intensive care or high dependency unit, or transfer to another facility.

† Maternal death or near-miss. Geographical areas in six western European countries (Belgium, Denmark, Italy, Spain, the Netherlands, the UK) did not collect data for WHO near-miss criteria.

‡ Multinomial mixed models adjusting for clustering at the country level.

§ Source of infection clinically, radiologically, or microbiologically confirmed.

¶ Women who had an abortion, ectopic, or molar pregnancy.

‖ Women who underwent childbirth (stillbirth or live birth).

At least one source of infection was identified for 79·7% of women (table 3). The most common sources of maternal infections were of the genital (endometritis and chorioamnionitis) or urinary tract, skin or soft tissues, respiratory tract, and abortion-related. The most common source of infection leading to complications or severe maternal outcomes were endometritis, skin or soft tissue, and abortion-related.

**Table 3:** Characteristics of maternal infections and management by severity group

		**All women (n=2850)**	**Less severe infections (n=1835)**	Infections with complications*(n=634)	**Infection-related severe maternal outcome**†**(n=381)**	**p value**‡
Primary source of infection identified§		2271 (79·7%)	1368 (74·6%)	579 (91·3%)	324 (85·0%)	<0·0001
Source of infection§						
	Urinary tract	632 (27·9%)	504 (36·8%)	69 (12·0%)	59 (18·2%)	..
	Endometritis	343 (15·1%)	178 (13·0%)	88 (15·3%)	77 (23·8%)	..
	Chorioamnionitis	338 (14·9%)	238 (17·4%)	66 (11·5%)	34 (10·5%)	..
	Skin or soft tissue	336 (14·8%)	105 (7·7%)	185 (32·2%)	46 (14·2%)	..
	Respiratory	204 (9·0%)	116 (8·5%)	21 (3·7%)	67 (20·7%)	..
	Abortion-related¶	193 (8·5%)	33 (2·4%)	115 (19·9%)	45 (13·9%)	..
	Bloodstream	115 (5·1%)	97 (7·1%)	7 (1·2%)	11 (3·4%)	..
	Peritonitis or abdominal cavity	69 (3·0%)	4 (0·3%)	27 (4·7%)	38 (11·7%)	..
	Gastrointestinal	63 (2·8%)	39 (2·9%)	11 (1·9%)	13 (4·0%)	..
	Breast	30 (1·3%)	22 (1·6%)	5 (0·9%)	3 (0·9%)	..
	CNS	10 (0·4%)	3 (0·2%)	1 (0·2%)	6 (1·9%)	..
	Other	197 (9·2%)	132 (10·6%)	42 (8·0%)	23 (7·1%)	..
Method of identification of the infection if source identified						
	Clinical examination alone	910 (40·1%)	526 (38·5%)	282 (48·7%)	102 (31·5%)	..
	Clinical examination and laboratory test	890 (39·2%)	648 (47·4%)	159 (27·5%)	83 (25·6%)	..
	Clinical examination and imaging	201 (8·8%)	85 (6·3%)	49 (8·5%)	67 (20·6%)	..
	Clinical examination, laboratory, test, and imaging	267 (11·7%)	107 (7·8%)	88 (15·2%)	72 (22·2%)	..
Complete set of vital signs recorded on day infection was suspected or confirmed		1821 (63·9%)	1100 (59·9%)	435 (68·6%)	286 (75·1%)	0·0093
Antimicrobials started the day of suspicion or diagnosis of infection‖		1875 (70·2%)	1198 (70·6%)	435 (71·6%)	243 (66·4%)	0·37
Antibiotics started the day of suspicion or diagnosis of infection‖		1843 (70·2%)	1165 (70·5%)	435 (71·7%)	243 (66·4%)	0·58
Sample for culture drawn at any time**		1269 (46·6%)	788 (46·0%)	280 (44·7%)	201 (52·8%)	0·19
Sample for culture drawn before administration of antibiotics		760/1177 (64·6%)	496/745 (66·6%)	165/254 (65·0%)	99/178 (55·6%)	0·044
Any microorganism identified by any method††		590 (21·2%)	360 (20·0%)	147 (25·6%)	101 (31·2%)	0·0017
Any positive culture of any body fluid**		579 (25·6%)	331 (24·2%)	133 (21·6%)	97 (26·1%)	0·011
All microorganisms identified by any methods‡‡						
	Bacteria	455 (77·1%)	257 (71·4%)	116 (87·2%)	82 (84·5%)	..
	Fungi	47 (8·0 %)	30 (8·3%)	6 (4·5%)	11 (11·3%)	..
	Parasite	94 (15·9%)	79 (21·9%)	7 (5·3%)	8 (8·2%)	..
	Virus	21 (3·6%)	13 (3·6%)	3 (2·3%)	5 (5·1%)	..
Additional management to control the source of infection§§						
	Vacuum aspiration	108 (4·0 %)	..	83 (13·0 %)	25 (7·0 %)	..
	Dilatation and curettage	160 (5·6%)	..	131 (20·7%)	29 (7·6%)	..
	Wound debridement	162 (5·7%)	..	136 (21·5%)	26 (6·8%)	..
	Drainage (incision, percutaneous, culdotomy)	153 (5·4%)	..	102 (16·0%)	51 (13·4%)	..
	Hysterectomy	55 (1·9%)	..	0	55 (14·4%)	..
	Laparotomy and lavage	201 (8·0%)	..	111 (17·5%)	90 (23·6 %)	..
	Other surgery	86 (3·2%)	..	64 (10·1%)	22 (5·8%)	..
Median length of stay in health facility, days (IQR)		5 (3–9)	5 (3–7)	7 (4–11)	9 (5–17)	<0·0001
Admission to intensive or high dependency care		355 (13·8%)	..	167 (27·7%)	188 (49·3%)	<0·0001

Data are n (%), n/N (%), or median (IQR) unless specified.

* Includes women who had an invasive procedure to treat the source of infection (vacuum aspiration, dilatation and curettage, wound debridement, drainage [incision, percutaneous, culdotomy] laparotomy and lavage, other surgery), admission to intensive care or high dependency unit, or transfer to another facility.

† Maternal death or near-miss. Geographical areas in six western European countries (Belgium, Denmark, Italy, Spain, the Netherlands, the UK) did not collect data on WHO near-miss criteria.

‡ Multinomial mixed models for percentages and linear model for logarithm (length of stay) adjusting for clustering at country level.

§ More than one source bpossible.

¶ Women who had an abortion, ectopic, or molar pregnancy.

‖ Same day or previous day after 1800 h.

** Includes culture drawn at entry in study or any time during stay in the facility.

†† Includes culture of any body fluid, microscopy, or specific test (eg, malaria, tuberculosis, HIV).

‡‡ Includes all organisms identified in women without inferring causation (when organism identified). Each woman could have more than one type of microorganism identified.

§§ More than one intervention possible.

Regarding the use of core practices for early identification and management of maternal infections, close to two-thirds of women had a complete set of vital signs recorded, and 70·2% received antibiotics or other antimicrobials the day of suspicion or diagnosis of the infection (table 3). Less than half of the women had samples drawn for culture at suspicion or confirmation of infection, and two-thirds of the samples were taken before the administration of antibiotics. Microorganisms were reported in 21·2% of the samples, bacteria being the most frequent (77·1%); they are reported here without inferring direct causality. There were no marked differences in use of this core set of practices for early identification and management of maternal infections across severity groups.

Table 4 shows pregnancy, maternal, and neonatal outcomes by pregnancy status at enrolment by infection severity group. Most women were discharged alive and had a live neonate who was discharged alive. Adverse pregnancy and neonatal outcomes increased with infection severity.

**Table 4:** Pregnancy, maternal, and neonatal outcomes by pregnancy status at entry in the study and severity group

		**All women (n=2850)**	**Maternal infection first suspected or diagnosed during pregnancy or labour**					**Maternal infection first suspected or diagnosed during post-partum or post-abortion**				
			All women who were pregnant or in labour (n=1335)	Less severe infections (n=1053)	Infections with complications* (n=147)	Infection-related severe maternal outcome† (n=135)	p value‡	All women puerperium (n=1515)	Less severe infections (n=782)	Infections with complications* (n=487)	Infection-related severe maternal outcome† (n=246)	p value‡
Pregnancy outcome§		..	..	..	..	..	<0·0001	..	..	..	..	<0·0001
	Still pregnant	662 (23·3%)	662 (49·7%)	549 (52·2%)	59 (40·1%)	54 (40·0%)	..	..	..	..	..	
	Abortion¶	322 (11·3%)	53 (4·0%)	26 (2·5%)	18 (12·3%)	9 (6·6%)	..	269 (17·9%)	71 (9·2%)	140 (28·7%)	58 (23·5%)	..
	Stillbirth	131 (4·6%)	51 (3·9%)	21 (2·0%)	11 (7·5%)	19 (14·1%)	..	80 (5·3%)	29 (3·7%)	21 (4·4%)	30 (12·2%)	..
	Livebirth	1730 (60·8%)	567 (42·5%)	455 (43·3%)	59 (40·1%)	53 (39·2%)	..	1163 (77·0%)	679 (87·1%)	326 (66·9%)	158 (64·2%)	..
Final mode of birth§‖		..	..	..	..	..	0·0007					<0·0002
	Vaginal birth	793 (44·3%)	301 (50·3%)	248 (53·9%)	25 (36·8%)	28 (39·4%)	..	492 (41·3%)	321 (46·6%)	106 (32·5%)	65 (36·7%)	..
	Caesarean section	998 (55·7%)	298 (49·7%)	212 (46·1%)	43 (63·2%)	43 (60·6%)	..	700 (58·7%)	368 (53·4%)	220 (67·5%)	112 (63·3%)	..
Maternal status at end of follow-up**		..	..	..	..	..	<0·0001	..	..	..	..	<0·0001
	Discharged alive	2775 (97·5%)	1302 (97·8%)	1050 (100%)	134 (91·8%)	118 (87·4%)	..	1473 (97·2%)	782 (100%)	477 (98·0%)	214 (87·0%)	..
	Transferred	45 (1·6%)	19 (1·4%)	..	12 (8·2%)	7 (5·2%)	..	26 (1·7%)	..	10 (2·0%)	16 (6·5%)	..
	Passed away	26 (0·9%)	10 (0·8%)	..	0	10 (7·4%)	..	16 (1·1%)	..	0	16 (6·5%)	..
Neonatal status at end of follow-up§††		..	..	..	..	..	<0·0001	..	..	..	..	0·095
	Discharged alive	1551/1834 (84·5%)	544/643 (84·6%)	436/517 (84·4%)	59/68 (86·8%)	449/58 (84·5%)	..	1007/1191 (84·5%)	623/699 (89·1%)	265/331 (80·0%)	119/161 (73·9%)	..
	Early neonatal death	67/1834 (3·7%)	23/643 (3·6%)	12/517 (2·3%)	5/68 (7·4%)	6/58 (10·3%)	..	44/1191 (3·7%)	19/699 (2·7%)	17/331 (5·1%)	8/161 (5·0%)	..
	Unknown	216/1834 (11·8%)	76/643 (11·8%)	69/517 (13·3%)	4/68 (5·9%)	3/58 (5·2%)	..	140/1191 (11·8%)	57/699 (18·2%)	49/331 (14·8%)	34/161 (21·1%)	..

Data are n (%) or n/N (%), unless specified. Percentages were calculated using available data.

* Includes women who had an invasive procedure to treat the source of infection (vacuum aspiration, dilatation and curettage, wound debridement, drainage [incision, percutaneous, culdotomy], laparotomy and lavage, other surgery), admission to intensive care or high dependency unit, or transfer to another facility.

† Maternal death or near-miss. Six western European countries (Belgium, Denmark, Italy, Spain, the Netherlands, the UK) did not collect data for WHO near-miss criteria.

‡ Multinomial mixed models adjusting for clustering at country level.

§ Includes data for multiple pregnancies.

¶ Women who had an abortion, ectopic, or molar pregnancy.

‖ Women who underwent childbirth (stillbirth or livebirth).

** Discharge from health facility, transfer outside the geographical area or death.

†† Newborns born alive, end of follow-up was at discharge from facility after birth, transfer outside the geographical area, death, or day 7 after birth if still hospitalised.

## Discussion

This is the first study, to our knowledge, to provide data for frequency and management of maternal infections requiring hospital management in a large number of LMICs and HICs, and across the continuum of pregnancy and post-pregnancy periods up to 42 days. The observed frequency of suspected or confirmed maternal infections was of 70·4 pregnant or recently pregnant women per 1000 live births. The burden of intrahospital severe outcomes related to maternal infections is high, with more than a third of women who had an infection developing severe maternal outcomes or requiring invasive procedures to treat the source of infection, admission to an intensive care unit or high dependency care, or transfer to another facility. Lack of adequate assessment of vital signs and delays in antimicrobial therapy were frequent.

Our results suggest that there are marked differences in frequency of infections and outcomes of maternal infections between LMICs and HICs. The burden of infection-related severe maternal outcomes is lower in HICs compared with LMICs, as previously described.^7^, ^8^, ^11^, ^12^ Although we found rates of infection-related severe maternal outcomes in HICs similar to those previously reported,^7^, ^8^, ^12^ rates in LMICs are much higher in our sample, particularly in upper-middle-income countries.^11^ The observed variation across countries could be related to use of different admission criteria or resources available to identify severe conditions or to manage in-patient women with infections across facilities, geographical areas, and countries. This difference in identification and management of women with infection could partly explain the higher burden of infectious morbidities in upper-middle-income countries than lower-middle-income countries, where facilities might have lower thresholds for admission of women with maternal infection or more resources to identify or treat complications compared with facilities in low-income countries.

Infections were the underlying cause of most intrahospital deaths attributed to other direct (eg, abortion-related) and indirect (eg, respiratory infection, meningitis) causes. Infections were also present in about a third of deaths attributed to other causes, in concurrence with previous findings in obstetric,^20^, ^21^, ^22^, ^23^ and general populations.^10^ These results suggest that the role of infections as an underlying or contributing cause of maternal deaths, across the continuum from pregnancy to post-partum or post-abortion, is higher than previously thought. As previously suggested, the inclusion of non-obstetric wards in our study might have led to an increase in identification of maternal deaths and near-miss cases related to indirect infections.^20^ This finding could also reflect a trend towards an increasing proportion of indirect causes of maternal deaths, although the contribution of infections as a direct cause of severe maternal outcomes remains high.^20^, ^24^ It is worth noting that the distribution of causes of intrahospital maternal deaths is close to the most recent estimates of causes of maternal mortality.^1^

The most common infections identified in this study were urinary tract infections, endometritis, chorioamnionitis, abortion-related infections, and skin and soft tissue, in line with previous studies.^2^, ^8^, ^25^ Several of the obstetric infections identified in our sample and associated with severe maternal outcomes, namely skin and soft tissue and abortion-related infections, are highly preventable. Good infection control measures are key for the prevention of infections after caesarean section, episiotomy, or other invasive procedures.^15^, ^16^, ^26^ In addition, prophylactic antibiotics are recommended to reduce infections due to caesarean section. However, data for coverage of prophylactic antibiotics for caesarean section suggest that its use is suboptimal across the world,^2^, ^27^ with wide variations across health facilities.^28^ Post-abortion infections are preventable through access to safe abortion, and prompt appropriate management of abortion-related complications.^16^

This study highlights important gaps regarding early identification and management of maternal infections in health facilities. A complete set of vital signs on the day of suspicion or diagnosis of infection was not reported for a third of women. Although most women received antimicrobials around the time of suspicion or diagnosis of the infection, about a third did not, and fewer than half had samples drawn for cultures before administration of antibiotics. In general, women with severe maternal outcomes had fewer invasive procedures to control the source of infection. Previous studies have also reported inadequate recognition and management of women with infection and sepsis in LMICs^20^, ^29^ and HICs,^8^, ^28^ including incomplete monitoring and delayed initiation of antibiotics. A substantial opportunity exists for improvement in early identification and prompt management of women with infections in health facilities, requiring more than just raising awareness.^19^ The use of quality improvement initiatives, including bundles, protocols, and checklists contribute to improving practices and outcomes. The use of trigger tools has shown an increase in the recording of vital signs and improved management.^17^ Timely completion of bundles of care (1 h and 3 h bundles) has also been associated with a reduction in adult mortality.^14^ These strategies should also contribute to better antimicrobial stewardship and strengthen efforts to minimise antimicrobial resistance.^13^, ^15^, ^30^

Our study is one of the few to report data for maternal infections in the continuum of pregnancy, childbirth, and the post-partum or post-abortion period, and across different severity groups. We sought to identify women with maternal infections by ensuring a good coverage of facilities within geographical areas, including participation of non-obstetric wards. The awareness campaign might have contributed to better identification of eligible cases. However, generalisability of results is limited to intrahospital outcomes and geographical areas similar to those included in the study. Comparisons with other studies reporting on the burden of infections and sepsis are limited by differences in case definitions, sources of infections considered, or stage of pregnancy included.^8^, ^25^ Temporality of organ dysfunction and infection was difficult to assess in our study, which might result in overestimating sepsis cases. However, as discussed in previous studies, diagnosing infection and attributing organ dysfunction to infection are often subjective.^30^ Reverse causality might also complicate this association in cases of multiple maternal complications—eg, infection and post-partum haemorrhage.^21^ A detailed description of strategies put in place to address potential sources of selection and measurement bias in our study is presented in the published protocol.^18^ We expected a minimal effect of geographical or seasonal clusters of infectious morbidities given the large number of countries distributed between the northern and southern hemispheres and that most of the cases would be genitourinary tract infections not subjected to seasonality. Our study did not evaluate infections not requiring hospital management and was not designed to cover maternal infection-related deaths in the community. Inclusion in the study was based on standard routine care in participating health facilities, including admission and diagnosis criteria, as well as collection of any laboratory, diagnostic, or other investigations. However, in women with complications or severe maternal outcomes, we would not have expected differing admission thresholds. This group is likely to have needed in-hospital management regardless of the admission criteria or resources available, in particular for women with infection-related near-miss who otherwise would have died if not treated in the facility. We did not collect the time of initiation of antimicrobials and therefore were not able to evaluate compliance with 3 h and 6 h sepsis bundles. The study design did not enable us to evaluate the long-term effects of maternal infections, including for example readmission or death after discharge, or infertility.^7^, ^8^

The frequency of infections among pregnant or recently pregnant women requiring management in health facilities is high. Our results suggest that contribution of direct and indirect infections to overall maternal deaths is greater than previously thought. There is a substantial opportunity to improve the prevention, early identification, and prompt management of women with infections in health facilities by implementing effective evidence-based practices.

Correspondence to: Dr Mercedes Bonet, Department of Sexual and Reproductive Health and Research, WHO, Geneva 27 CH-1211, Switzerland bonetm@who.int

## Data sharing

The data used for this analysis can be made available upon reasonable request, in accordance with the GLOSS research group data sharing policy and WHO policy of data use and data sharing. For further information, contact the corresponding author.
