# Supplementary material for: Frequency and management of maternal infection in health facilities in 52 countries (GLOSS): a 1-week inception cohort study
Source: Lancet Glob Health. 2020 Apr 27;8(5):e661–71. doi: 10.1016/S2214-109X(20)30109-1 (PMC7196885; doi:10.1016/S2214-109X(20)30109-1)
Supplement: Supplementary appendix [file mmc1.pdf]

THE LANCET
Global Health

Supplementary appendix

This appendix formed part of the original submission and has been peer reviewed. We post it as supplied by the authors.

Supplement to: The WHO Global Maternal Sepsis Study (GLOSS) Research Group. Frequency and management of maternal infection in health facilities in 52 countries (GLOSS): a 1-week inception cohort study. *Lancet Glob Health* 2020; **8:** e661–71.

Supplementary materials

Table S1. Reference list of infections* associated with systemic repercussions during pregnancy, childbirth, post abortion and postpartum period (modified from ICD-MM, the WHO Application of ICD-10 to deaths during pregnancy, childbirth, and the puerperium)

Pregnancy-related infection (ICD-MM Group 4)

- Acute pyelonephritis
- Infection of amniotic sac and membranes (amnionitis, chorioamnionitis, membranitis, placentitis)
- Retained products of conception
- Endometritis, endomyometritis
- Pelvic abscess
- Uterine microabscess or necrotizing myometritis
- Necrotizing fasciitis
- Necrotizing vulvitis
- Infection of obstetric surgical wound (caesarean section, perineal repair)
- Episiotomy infection or dehiscence
- Other infection of genital tract following delivery (cervicitis, vaginitis following delivery, genital tract laceration)
- Pyrexia of unknown origin following delivery
- Infections of breast associated with childbirth (abscess of the nipple, abscess of the breast, subareolar abscess, mastitis, lymphangitis of breast)
- Tetanus

Maternal infectious and parasitic diseases classifiable elsewhere but complicating pregnancy, childbirth and the puerperium

- Pneumonia
- Other pulmonary infections (mycoplasma, legionella)
- Acute viral infections (influenza, H1N1, herpes with systemic repercussion, varicella, acute infectious hepatitis, Encephalitis, dengue, chikungunya, yellow fever, other haemorrhagic fever
- Malaria
- Complicated tuberculosis
- Listeriosis
- Leptospirosis
- Rickettsioses (scrub typhus, murine typhus)

Exclusion criteria. Women presenting the following conditions were excluded, unless they present with systemic repercussion due to infection:

- Any non-severe, localized, uncomplicated infection
    - Vaginosis, candidiasis
    - Lower tract urinary infection
    - Fungal infections of the skin (athlete's foot, jock itch, ringworm, and yeast infections)
    - Otitis
    - Pharyngitis
    - Herpes simplex, herpes zoster (shingles)
- Any uncomplicated chronic infection
    - Sexually transmitted infections (gonorrhoea, syphilis, trichomonas, chlamydia, hepatitis, HIV)
    - Tuberculosis
- Any colonization (presence of microorganisms without clinical signs/symptoms)
    - Known GBS vaginal, urethral and/or rectal colonization
    - Asymptomatic bacteriuria
    - Known oropharyngeal colonization
- Any iatrogenic hypothermia/hyperthermia (e.g. related to epidural, thyroid storm, prostaglandin administration) during hospital stay
- Use of any prescription of prophylactic antibiotics (e.g. for GBS colonization, after caesarean section, manual removal of the placenta, vaginal delivery)

*study eligibility was not limited to those

Table S2. Characteristics of participating facilities

Characteristics		n (%)
Public facility (yes)		417 (78·5%)
Location		
	Urban	514 (80·7%)
	Peri-urban	80 (12·6%)
	Rural	43 (6·8%)
Level		
	Tertiary	220 (34·5%)
	Secondary	305 (47·9%)
	Primary	220 (34·5%)
Country income level*		
	Low income	82 (11·5%)
	Lower-middle income	200 (28·1%)
	Higher-middle income	126 (17·7%)
	High income	305(42·8%)
Maternity exclusive hospital (yes)		95 (14·6%)
Median number of births/year in 2016 (Q1-Q3)		1993 (861-4211)
Number of births per year in 2016		
	<1000	137 (26·7)
	1000-2499	156 (30·4)
	2500-4499	103 (20·1)
	>4500	117 (22·8)
Median number of births during identification week (Q1-Q3)		40 (16-78)
Adult intensive care or high dependency unit (yes)		540 (84·8%)

*using the 2018 World Bank classification (https://datatopics.worldbank.org/worlddevelopment-indicators/theworld-by-income-and-region.html)

Table S3: Distribution of underlying causes of maternal deaths

	Total maternal deaths		Maternal deaths that occurred during the week of identification	
	Primary cause of maternal death[b] (n=46)	Infection-related maternal deaths[a,b,c] (n=26)	Primary cause of maternal death[b] (n=39)	Infection-related maternal deaths[a,b,c] (n=19)
Obstetric haemorrhage	9 (20%)	2 (8%)	9 (23%)	2 (11%)
Hypertensive disorder	8 (17%)	1 (4%)	7 (18%)	0 (0%)
Infection/Sepsis	7 (15%)	7 (27%)	6 (15%)	6 (32%)
Other direct	1 (2%)	1 (4%)	1 (3%)	1 (5%)
Abortion	5 (11%)	5 (19%)	3 (8%)	3 (16%)
Any indirect cause	11 (24%)	8 (31%)	9 (23%)	6 (32%)
Unknown	5 (11%)	2 (8%)	4 (10%)	1 (5%)

[a] Infection is underlying or contributing cause of maternal death; [b] Includes all maternal deaths reported in the study: 20 maternal deaths not related to infections; [c] Seven maternal deaths occurred after the identification week.

Table S4: Distribution of organ dysfunction by system

	Severe maternal outcomes related to infections (n=381)	Infection-related near-miss (n=355)	Infection-related maternal death (n=26)
Cardiovascular	160 (42.0%)	139 (39.1%)	21 (80.8%)
Respiratory	150 (39.4%)	130 (36.6%)	20 (76.9%)
Renal	66 (17.3%)	57 (16.1%)	9 (34.6%)
Coagulation	105 (27.6%)	97 (27.3%)	8 (30.8%)
Hepatic	46 (12.1%)	42 (11.8%)	4 (15.4%)
Neural	34 (8.9%)	25 (7.0%)	9 (34.6%)
Uterine	79 (20.7%)	75 (21.1%)	4 (15.4%)
None	1 (0.3%)	0 (0.0%)	1 (3.8%)
Multiple organ system dysfunction	143 (37.5%)	122 (34.4%)	21 (80.8%)
